# Networks of care for the modern adolescent

**DOI:** 10.1017/S003329172400237X

**Published:** 2024-12

**Authors:** Simon R. White, Emma Soneson, OxWell Study Team, Mina Fazel

**Affiliations:** 1 Department of Psychiatry, University of Cambridge, Cambridge, UK; 2 MRC Biostatistics Unit, University of Cambridge, Cambridge, UK; 3 Department of Psychiatry, University of Oxford, Oxford, UK

**Keywords:** adolescence, adolescents, CAMHS, children and young people, mental health, schools, services, support

## Abstract

**Background:**

At a time of increased demand for specialist mental health services, a more nuanced understanding of how adolescents navigate systems of care and support is essential. We mapped ‘networks of care’ to explore patterns of mental health help-seeking alongside the perceived helpfulness of support accessed.

**Methods:**

We examined data from 23 927 adolescents aged 11–18 years who participated in the 2023 OxWell Student Survey, an English school-based, repeated cross-sectional survey of mental health and wellbeing. Students self-reported past-year access to 18 types of support across informal (e.g. friends and family), semi-formal (e.g. school and charities), and formal (e.g. health and social care) domains, alongside how helpful they found the support. We used a network approach to explore interconnections between sources of support accessed and perceived helpfulness.

**Results:**

One in four (27.0%, 6449/23927) adolescents reported past-year access to mental health support, of which 56.7% (3658/6449) reported accessing multiple types. Informal networks were the most commonly accessed (23.1%, 5523/23927), followed by semi-formal (9.7%, 2317/23927) and formal (6.8%, 1623/23927) supports. Informal sources had high acceptability, with around 80–90% reporting them as helpful, whereas child and adolescent mental health services (CAMHS), helplines, and online supports were perceived to be the least helpful. The networks also identified groups who might not be optimally served by current systems, including gender diverse adolescents and adolescents who found mental health support from their parents unhelpful.

**Conclusions:**

Adolescents are accessing mental health support across informal, semi-formal, and formal sources of care. Services can no longer be developed, delivered, or evaluated in isolation from these networks.

## Introduction

There is a pressing need to better understand how, when, and from whom adolescents seek mental health support. Adolescent mental health difficulties represent a substantial public health challenge with serious short, medium, and long-term implications for individuals, families, and society (Beecham, 2014; Clayborne, Varin, & Colman, 2019; Sellers et al., 2019; Snell et al., 2013). Timely access to care and support may help mitigate some of these consequences by equipping adolescents and their families with the skills and resources needed to manage mental health difficulties and reduce associated impacts (Dodge et al., 2015; McGorry & Mei, 2018). However, abundant evidence demonstrates that access to support is sub-optimal amongst this group (Costello, He, Sampson, Kessler, & Merikangas, 2014; Ford, Hamilton, Meltzer, & Goodman, 2007; Mandalia et al., 2018; Merikangas et al., 2011; Schnyder et al., 2019; Shidhaye, 2023), suggesting that a better understanding of adolescents' perspectives on the accessibility and acceptability of mental health support may help to more effectively target and tailor services and interventions (MacDonald, Fainman-Adelman, Anderson, & Iyer, 2018).

Specialist mental health services and other healthcare providers have been the traditional focus of research on mental health help-seeking (Heerde & Hemphill, 2018). However, it is becoming increasingly clear that many school-aged adolescents rely on support outside of these arenas (Duong et al., 2021; Heerde & Hemphill, 2018; Pretorius, Chambers, & Coyle, 2019), most notably at school and from informal support systems including friends and family. English national survey data demonstrate that teachers and school staff are the most accessed professional source of mental health support for adolescents with and without psychiatric diagnoses (Mathews, Ford, White, Ukoumunne, & Newlove-Delgado, 2024) and additionally highlight the importance of informal support (Mandalia et al., 2018). The self-harm literature also demonstrates the value of informal support, with adolescents who have self-harmed reporting having sought help from friends and family more often than any other source (Fortune, Sinclair, & Hawton, 2008; Geulayov et al., 2022). Therefore, a lens that encompasses the breadth of the many sources and settings of adolescent mental health support is essential (Duong et al., 2021).

Exploration of adolescents' perceptions of the helpfulness of support accessed is also required, not only to further our understanding of the barriers and facilitators of help-seeking (e.g. Anderson, Howarth, Vainre, Jones, & Humphrey, 2017; Radez et al., 2020), but also because these perceptions are associated with treatment outcomes (Brown, Ford, Deighton, & Wolpert, 2014). Assessments of satisfaction within child and adolescent mental health services (CAMHS) and other formal services, whilst valuable, often have substantial biases, as measurement typically occurs at the end of treatment and thereby excludes those who have not engaged for the duration (Plaistow et al., 2014). Insight from these less-engaged groups is particularly relevant in service design, and community-based samples have substantial potential for better understanding how adolescents perceive different avenues of support. Studies using such samples present a varied picture, though generally suggest that adolescents often view informal support as more helpful than formal support. For example, the English national survey found that 61–73% of children and adolescents with mental health difficulties who had accessed professional support services found them helpful, compared with 85% for support from friends and family (Mandalia et al., 2018), and an earlier OxWell analysis found that adolescents who have self-harmed preferred informal support over formal services, school-based support, or online support (Geulayov et al., 2022).

A more nuanced understanding of adolescents' help-seeking behaviors and experiences can help to improve the range of services and quality of care provided (Fazel & Hoagwood, 2021; Howarth et al., 2019; MacDonald et al., 2018; Persson, Hagquist, & Michelson, 2017; Plaistow et al., 2014). This is of particular relevance in spheres of provision including informal or school-based supports, which adolescents seem most likely to access but where objective measures such as service use data, patient-reported outcome measures, and clinical assessments are not readily available. At a time where demand for specialist mental health services is at an all-time high (Thomas, Schroder, & Rickwood, 2021), and there are an increasing number of system-wide changes being considered and implemented to improve access to mental health support (Department of Health & Department for Education, 2017; MacDonald et al., 2018), integrating adolescent voices is essential. These transformations will likely be more effective and meaningful if informed by adolescent perspectives on how they navigate systems of care and what support they perceive to be most valuable. To advance our understanding of these key issues, we analysed data from a large school-based survey to explore patterns of help-seeking amongst school-aged adolescents (‘networks of care’) and assessed how helpful they find various types of support.

## Methods

### Data source

This study used data from the secondary school section of the 2023 OxWell Student Survey (Mansfield et al., 2021), a self-report repeated cross-sectional survey examining the health and wellbeing of students in English secondary schools and further education colleges. The age range for the present analyses, 11–18 years, was designed around the English school system and represents a subset of the ages included in many of the definitions of adolescence (Sawyer, Azzopardi, Wickremarathne, & Patton, 2018; World Health Organization, 2019). In the OxWell study, schools are recruited directly by local authorities and enroll students using (1) a parental opt-out model and student assent (student age <16 years) or (2) students' informed consent only (student age ⩾16 years). The survey is administered during the school day and does not collect identifiable data about students to increase the diversity and representativeness of the sample, enable a parental opt-out model to ensure maximum participation, and encourage more accurate and honest responses (Mansfield et al., 2021). Responses were collected February–March 2023.

Youth co-production is an essential component of the OxWell survey and has included four youth advisors and 12 different school-aged students. We have sought advice at all stages, from question design to survey administration, interpretation of results, and dissemination of findings. Youth involvement for this specific study included ensuring that the list of types of mental health support was as comprehensive as possible and that the questions were worded in an appropriate and comprehensible manner. Furthermore, a young people's advisory group (YPAG) was also consulted to help us interpret the findings.

The 2023 OxWell Student Survey was approved by the University of Oxford Research Ethics Committee (reference: R62366/RE014).

### Measures

Our main outcomes were past-year access to mental health support (including number and type(s) of support accessed) and the perceived helpfulness of that support. Past-year access was assessed with the following question: ‘In the last 12 months, have you tried to ask for support for a mental health problem from the following [support category]’, with 18 options grouped across three categories: (1) ‘informal’ support (friends or family); (2) ‘semi-formal’ support (school services, online/helpline and other services (e.g. charities)); and (3) ‘formal’ support (statutory services including health and social care). The categories had six, eight, and four options respectively (with two additional free text options excluded). Upon selecting a type of support, participants were presented with two additional questions. The first asked about the status of their help-seeking, including whether they were currently accessing support, had previously accessed support, had not been offered support/had been turned away, or had changed their mind before accessing that support. We limit our analyses to the first two categories (‘current’ or ‘previous’ support), though provide full responses in online Supplementary Fig. S1. The second question asked about how helpful participants perceived that support to be, with answer categories ‘not helpful at all’, ‘not helpful enough’, ‘just about helpful enough’, ‘quite helpful’, and ‘very helpful’. For the purpose of these analyses, we collapsed these into ‘not helpful’ (comprising the first two categories) and ‘helpful’ (comprising the latter three categories).

Additional variables of interest included gender (‘male’, ‘female’, ‘other’ [with an option to self-describe], and ‘prefer not to say’); ethnic group (presented as 18 options and then grouped according to the 2021 Census Classification 6a [Census, 2021, 2023]); year group (7–13); self-identified past year mental health difficulties; and score on the 11-item Revised Children’s Anxiety and Depression Scale (RCADS-11) (Radez et al., 2021). Due to the sub-optimal nature of our gender question (which asked for gender but presented responses associated with sex), we worked with three trans and gender diverse (GD) young people to understand how to best use our data and jointly decided to categorize responses into boy, girl, GD, and gender non-disclosing (GND) (Soneson et al., In press). Due to sample size considerations and similarities in responses to later survey questions related to *concerns* about gender identity (Soneson et al., In press), we grouped GD and GND adoelscents into one ‘GD/GND’ category. Experience of self-identified past-year mental health difficulties was determined by an answer of ‘yes – in the past 12 months’ to the question ‘Do you think you've had a mental health problem that has affected your daily life?’ (other response options: ‘no’, ‘yes – more than a year ago’, ‘prefer not to say’). The RCADS-11 score was calculated using mean imputation that allowed for up to 1 missing item per subscale (anxiety and depressive symptoms), and we used cut-points published by Radez et al. (2021) to categorize adolescents as having elevated depression and anxiety symptoms; specifically ⩾9 for boys, ⩾14 for girls, and ⩾14 for GD/GND adolescents (*N.B.* there are no established cut-points for this last group, so we used the more conservative cut-point). The full OxWell variable guide is available at osf.io/bwech.

### Analysis

For all analyses, we subsetted our data to exclude students who had completely missing data for the questions on mental health support and all subsequent questions, under the assumption that these students did not reach the relevant page of the survey (see *Demographics*). Our analyses comprised two main parts: a hurdle model and network diagrams (including ‘anchored’ networks).

#### Hurdle model

**Figure 1.**
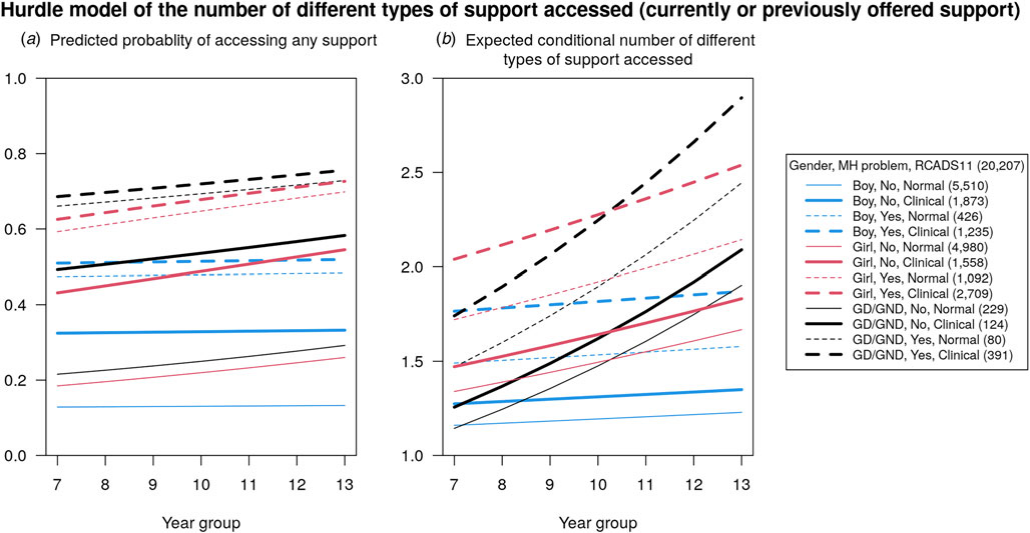
**Hurdle model estimating the number of past-year types of mental health support accessed.** Hurdle models consist of two components estimated simultaneously: a probability of having a zero or non-zero count (i.e. the probability of accessing at least one type of support, the ‘hurdle’) modelled using a logistic regression, and (if non-zero) the expected count modelled using a Poisson regression. The (a) predicted probabilities of having a non-zero count and (b) expected number of different types are shown for 12 sub-groups across year groups. Not all 12 groups are statistically significantly different (this information is not presented on the plots for clarity). Note: 3041 participants with unknown self-identified past-year mental health difficulties are omitted. See online Supplementary Table S2 for model details (including model coefficients). The expected number shown in (b) is conditional on ‘jumping the hurdle’, hence the *y*-axis starts at one.

The hurdle model aimed to estimate (1) the predicted probability of accessing any support (i.e. a non-zero number) and (2) for those who have sought support, the expected number of different types of support accessed. The hurdle model has two components: (1) a binary logistic regression and (2) a Poisson regression (truncated to be one or more, i.e. to exclude zero), which are estimated together. The components have a natural interpretation for those who accessed either *no* support or *some* support (and the effects of included regression covariates). The logistic regression makes direct inference on characteristics associated with accessing support. The Poisson count component is more nuanced, based on a truncated distribution and often with many fewer participants; we present a graphical representation (Fig. 1) that more clearly conveys the model fit but does not address the uncertainty (see online Supplementary Table S2).

#### Network diagrams

We use network diagrams to create data visualizations of adolescents' help-seeking patterns. Each network has three sample sizes: ‘*n*’ (total subsample analysed), ‘*v*’ (for *vertices*: all participants who accessed ⩾1 type of past-year support), and ‘*e*’ (for *edge counts*: all participants who accessed ⩾2 types of past-year support). Across all networks, it is important to stress that it is not possible to determine any temporal patterns due to the cross-sectional nature of the data and structure of the questions.

The size of each node corresponds to the number of participants with current or previous past-year access to a given type of support, excluding non-responders to the question on support status. The shape of the node (circle, square, hexagon) corresponds to the group of support types (classified as ‘informal’, ‘semi-formal’, and ‘formal’ respectively). The colour of the node corresponds to how many found that type of support helpful, as a proportion of all participants who indicated they had accessed the support and responded to the question on helpfulness (i.e. denominator excludes non-response). The thickness of the lines between pairs of nodes (i.e. edges) correspond to the proportion of participants that have reported past-year access to both types of support.

**Figure 2.**
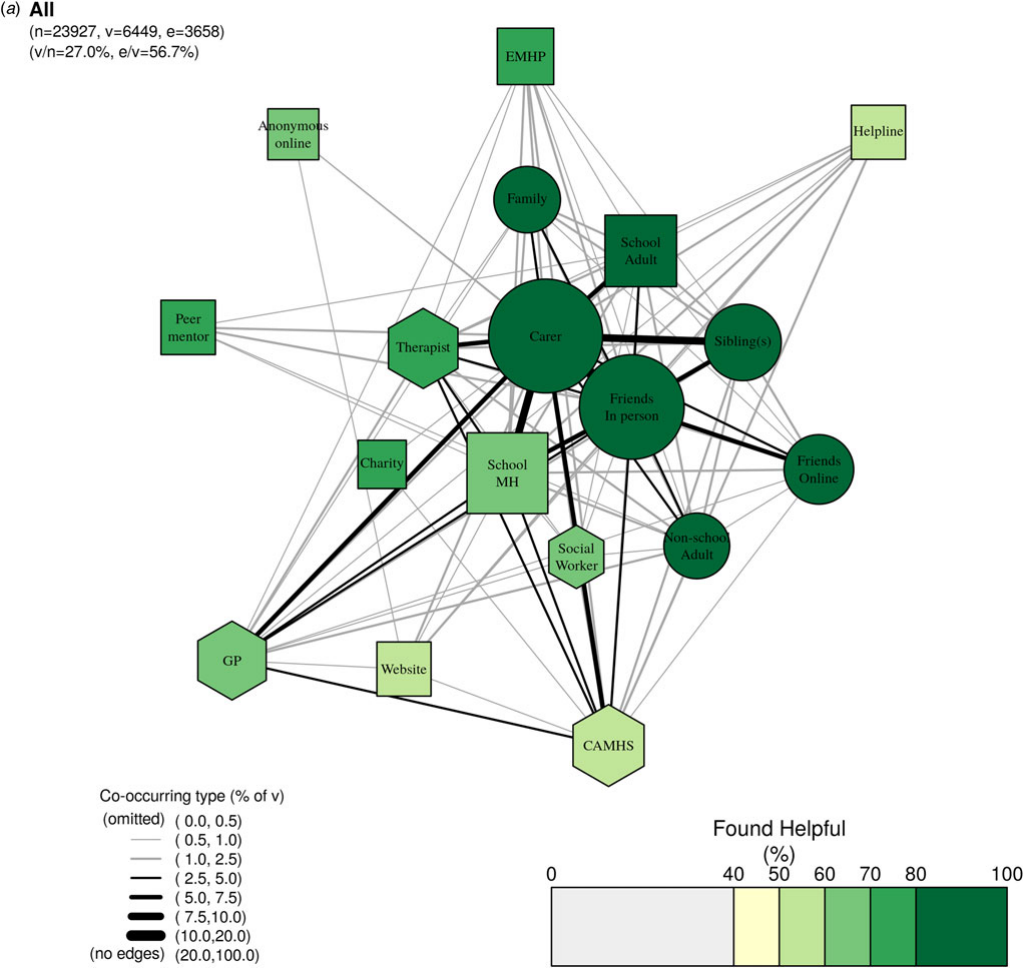
**Network of care for all participants.** Within the network, node size corresponds to the absolute number of participants who accessed each type of support in the past year; edge thickness corresponds to the proportion of participants who accessed both types of support; node colour corresponds to the proportion of participants who found each type of support helpful; and node shape corresponds to our grouping of support types where circles are informal supports, squares semi-formal, and hexagons formal. Legend for short labels: Carer: parent, step-parent, or carer; Sibling: sibling(s); Family: someone else in your family; Friends In Person: friend(s), mainly known in person; Friends Online: friend(s), mainly known online; Other Adult: an adult outside of school (at a sport club, another parent, family friend); School MH: school nurse/counsellor/other pastoral staff at school; EMHP: Education Mental Health Practitioner; School Adult: another adult at school; Peer: a peer mentor at school; Charity: support service given by a charity; Helpline: a telephone/text helpline; Website: website or online forum; Anonymous Online: an anonymous user on an online platform/chatroom/forum/server; GP: GP (family doctor); Social worker: social worker; CAMHS: NHS Child and Adolescent Mental Health Services; Therapist: private counsellor/therapist.

The position of the nodes was determined using all participants (i.e. Fig. 2); this is a so-called force-directed layout where more-connected nodes are closer together. Technically, each network should have a new layout of the nodes; however, to facilitate comparison across diagrams, we keep the layout constant and rely upon the node size and edge style to illustrate patterns within each network. Node size (reflecting the absolute number of participants accessing a given type of support) is comparable across diagrams, whilst edge style (reflecting the proportion of participants who accessed two given types of support) should be interpreted relative to the number of participants using two or more services (‘*v*’) and varies across diagrams.

#### ‘Anchored’ networks

Viewing each network as a pattern of help-seeking, we can ask particular questions by ‘anchoring’ on a specific support type. To anchor a network, we subsetted to participants who accessed a given type of support and responded to the question about helpfulness (by definition, *n* *=* *v* in an anchored network, since all participants must have engaged with the anchored support type). We differentiate these networks by the colour of the anchored node, with dark blue nodes representing networks of adolescents who found the focal support type *helpful* and light blue nodes representing networks of adolescents who found the focal support type *not helpful.*

## Results

### Demographics

The 2023 OxWell Student Survey collected 34 245 responses from students in school years 7–13. We excluded 4974 students who did not assent/consent or minimally engage with the survey (Mansfield et al., 2021) and 5344 participants who, likely due to time constraints, did not reach the stage of the survey that asked about mental health support (see online Supplementary Table S1 for a description of this group). We therefore consider a subsample of 23 927 participants from 78 secondary schools and further education colleges. In the final sample, 51.1% of students were girls, 43.5% boys, and 4.4% GD/GND individuals, with a median age of 14 years (IQR 12–15). In terms of ethnicity, 53.4% were White, 14.5% were Asian/Asian British, 5.5% were from Mixed/Multiple ethnic groups, 4.5% were Black/Black British/African/Caribbean, and 3.9% were from ‘Other’ ethnic groups (18.2% did not report). In total, 27.0% reported past-year access to at least one type of mental health support, including 42.7% of those with elevated depression and anxiety.

### Hurdle model for counts

Figure 1 presents inference from the two-part hurdle model, which generally confirms findings in Table 1. Adolescents with self-identified past-year mental health difficulties and those with elevated depression and anxiety symptoms were most likely to report past-year access to mental health support, with a higher probability amongst girls and GD/GND adolescents compared with boys. For girls and GD/GND adolescents, probability of past-year support generally increased as year group increased. Expected counts of support accessed generally followed a similar pattern, with a particularly high expected count for GD/GND adolescents relative to girls and boys within the older age ranges.

**Table 1.** Mental health support accessed overall and by gender, year group, ethnicity, and RCADS-11

		Types of mental health supports accessed			
Responder descriptive		0	1	2–18	Total
All		*17 478*	2791	3658	*23 927*
Gender	Boy	8554	921	944	*10 419*
	Girl	8143	1671	2419	*12 233*
	GD/GND^a^	624	172	265	1061
	No response	157	27	30	214
Ethnicity^b^	Asian/Asian British	2694	357	410	3461
	Black/Black British/African/Caribbean	858	113	112	1083
	Mixed/Multiple ethnic groups	900	172	245	1317
	White	9009	1580	2191	*12 780*
	Other ethnic group	735	82	117	934
	No response	3282	487	583	4352
Year group^c^	Year 7	3825	527	623	4975
	Year 8	3633	545	634	4812
	Year 9	3424	497	630	4551
	Year 10	2385	362	536	3283
	Year 11	2118	448	597	3163
	Year 12	1285	245	399	1929
	Year 13	808	167	239	1214
RCADS-11 (by gender)^d^	Normal	*11 152*	1139	1252	*13 543*
	Boy, clinical (⩾9)	2410	530	630	3570
	Girl, clinical (⩾14)	2529	915	1483	4927
	GD/GND, clinical (⩾14)^e^	274	113	210	597
	No response for gender and/or RCADS-11	1113	94	83	1290

*Notes:* (a) For participant self-reported gender we have combined the gender diverse (GD) category with the gender non-disclosing (GND) category (see Methods); (b) we report ethnicity by ONS ethnic group classification 6a; (c) starting the survey was dependent on the year group question being answered and so there are no missing data for this question; (d) we present mean-imputed RCADS-11 scores, allowing for up to two missing items (⩽1 per sub-scale); (e) the RCADS-11 adolescent-report only has clinical/non-clinical thresholds for boys and girls; we have used a threshold of ⩾14 for GD/GND participants.

### Networks of care

‘Networks of care’ illustrate how adolescents navigate the complex system of supports available to them, as well as their perceptions of the helpfulness of those supports. Table 1, online Supplementary Figs S1 and S2, and Table S3 summarize the underlying data.

Figure 2 presents the network for all participants. Reflecting the hurdle model, the majority (56.7%; 3658/6449) of participants who reported having accessed past-year mental health support had accessed more than one type.

**Figure 3.**
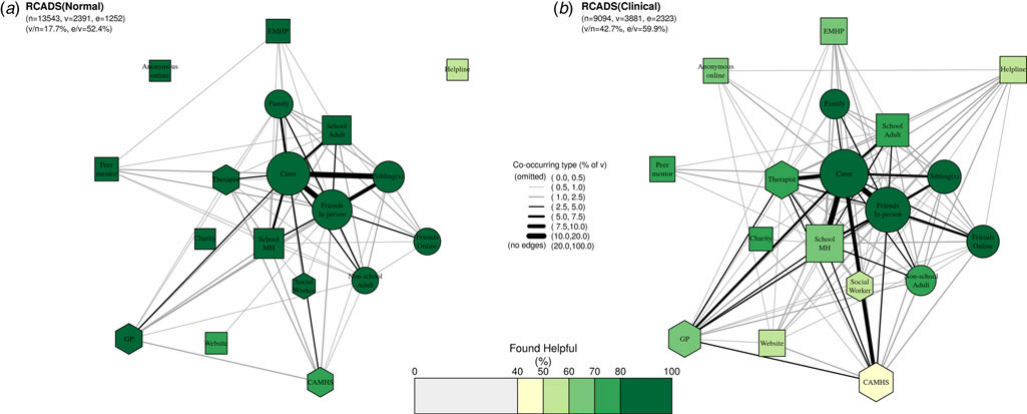
**Networks of care for (a) participants with depression and anxiety symptom scores in the ‘normal’ range (RCADS-11) and (b) participants with elevated depression and anxiety symptoms.** Within each network, node size corresponds to the absolute number of participants who accessed each type of support in the past year; edge thickness corresponds to the proportion of participants in the respective subgroup who accessed both types of support; node colour corresponds to the proportion of participants who found each type of support helpful; and node shape corresponds to our grouping of support types where circles are informal supports, squares semi-formal, and hexagons formal. Legend for short labels: Carer: parent, step-parent, or carer; Sibling: sibling(s); Family, someone else in your family; Friends In Person: friend(s), mainly known in person; Friends Online: friend(s), mainly known online; Other Adult: an adult outside of school (at a sport club, another parent, family friend); School MH: school nurse/counsellor/other pastoral staff at school; EMHP: Education Mental Health Practitioner; School Adult: another adult at school; Peer: a peer mentor at school; Charity: support service given by a charity; Helpline: a telephone/text helpline; Website: website or online forum; Anonymous Online: an anonymous user on an online platform/chatroom/forum/server; GP: GP (family doctor); Social worker: social worker; CAMHS: NHS Child and Adolescent Mental Health Services; Therapist: private counsellor/therapist.

Figure 3 presents networks for participants with elevated symptoms of anxiety and depression (Fig. 3(b)) and those without (Fig. 3(a)). There were stark differences between those with and without elevated symptoms in terms of the proportion who had accessed any support (i.e. v/n; 42.7% *v.* 17.7%, respectively), but, interestingly, less difference in terms of the proportion accessing multiple types of support (i.e. e/v; 59.9% *v.* 52.4%).

All three of the networks demonstrate that informal systems are the core source of support, in terms of how many adolescents report past-year support (node size) as well as the perceived helpfulness of the support (node colour). Of all participants, 23.1% (5523/23927) reported having accessed at least one type of informal support, most commonly parents/carers (13.9%, 3316/23927) and friends in-person and/or friends online (12.1%, 2895/23927). Friends, siblings, parents/carers, and other family members were also viewed favourably, with 87.4–91.1% finding these individuals helpful. Patterns were generally similar for those with elevated symptoms of anxiety and depression, of whom 36.2% (3294/9094) reporting having accessed at least one type of informal support. In total, 21.0% (1910/9094) reported past-year support from parents/carers and 19.2% (1749/9094) from friends in-person and/or friends online. Despite elevated symptoms, these adolescents also perceived informal support to be the most helpful of the three categories.

In total, 9.7% (2317/23927) of participants reported having accessed at least one type of semi-formal support, most commonly from school mental health teams (4.7%, 1128/23297) and other adults at school (3.0%, 723/23927). Within this category, other adults at school were perceived to be the most helpful (81.8% found them helpful), followed by peer mentors (74.1%). Compared with school-based support, other semi-formal supports, such as online and helpline support, were far less commonly accessed (each accessed by <1% of adolescents) and were viewed less favourably (just over half found these supports helpful). Patterns were generally similar for those with elevated symptoms of anxiety and depression, of whom 17.1% (1551/9094) reported having accessed at least one type of semi-formal support. In total, 8.8% (796/9094) of these adolescents reported past-year support from school mental health teams, 4.9% (450/9094) from other adults at school, and many fewer from online sources (1.7%, 157/9094) or helplines (1.8%, 168/9094). Perceived helpfulness again reflected findings from the overall sample, with other adults at school perceived to be most helpful (77.3%) and online and helpline support as the least helpful (53.5% and 54.2%, respectively).

Mental health support from formal institutions such as health and social care services was less commonly accessed, with 6.8% (1623/23927) of participants reporting having accessed at least one type of formal support. Access was approximately equal across CAMHS (2.9%, 701/23927), private counsellors/therapists (2.7%, 642/23927), and GPs (2.6% 602/23927). Formal supports were generally perceived as less helpful than other supports; for example, 55.5% of all participants who had accessed CAMHS found it helpful. This pattern was particularly evident for participants with elevated depression and anxiety symptoms, who were more likely to have accessed formal support (12.6% [1149/9094] *v.* 6.8% [1623/23927] of the full sample) but less likely to report finding it helpful. For them, CAMHS ranked amongst the lowest, with only 49.8% rating it as helpful.

**Figure 4.**
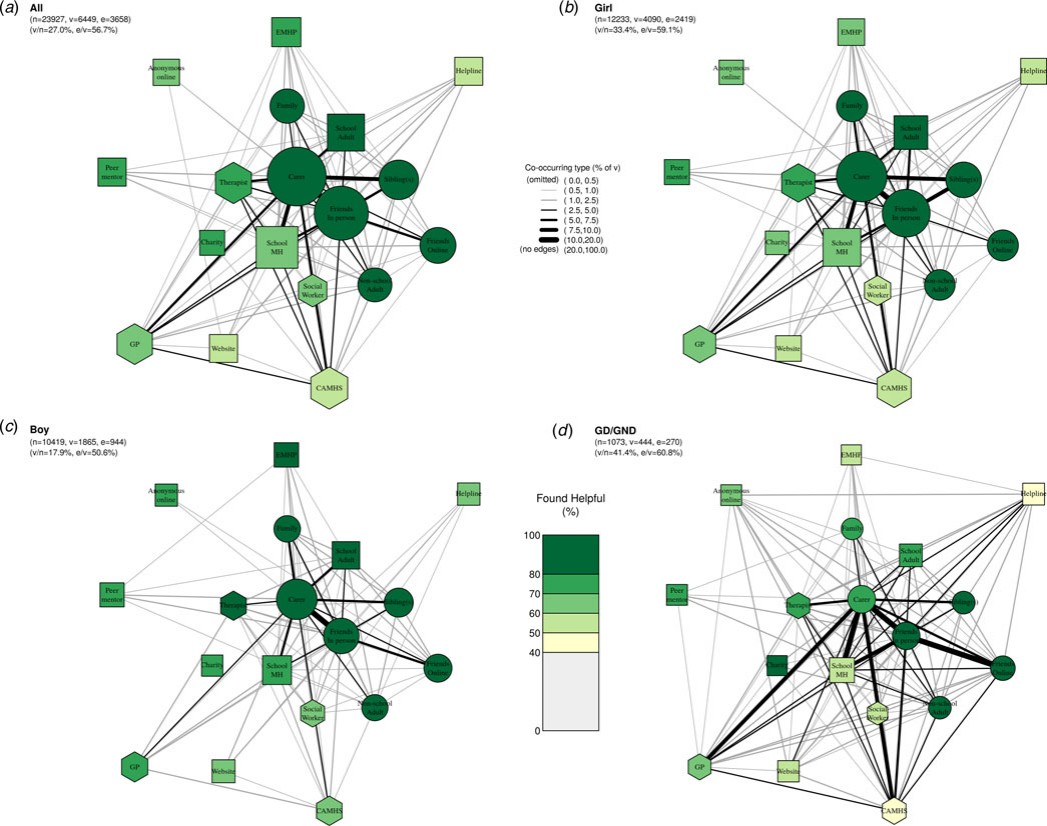
**Networks of care for (a) all participants (repeated from Fig. 2(a)); (b) girls; (c) boys; and (d) GD (gender diverse)/GND (gender non-disclosing) adolescents**. Within each network, node size corresponds to the absolute number of participants whoaccessed each type of support in the past year; edge thickness corresponds to the proportion of participants in the respective subgroup whoaccessed both types of support; node colour corresponds to the proportion of participants who found each type of support helpful; and nodeshape corresponds to our grouping of support types where circles are informal supports, squares semi-formal, and hexagons formal. Legend for short labels: Carer: parent, step-parent, or carer; Sibling: sibling(s); Family: someone else in your family; Friends In Person: friend(s), mainly known in person; Friends Online: friend(s), mainly known online; Other Adult: an adult outside of school (at a sport club, another parent, family friend); School MH: school nurse/counsellor/other pastoral staff at school; EMHP: Education Mental Health Practitioner; School Adult: another adult at school; Peer: a peer mentor at school; Charity: support service given by a charity; Helpline: a telephone/text helpline; Website: website or online forum; Anonymous Online: an anonymous user on an online platform/chatroom/forum/server; GP: GP (family doctor); Social worker: social worker; CAMHS: NHS Child and Adolescent Mental Health Services; Therapist: private counsellor/therapist.

Figure 4 presents networks stratified by gender. Reflecting findings from the hurdle model (Fig. 1), these networks demonstrate that girls and GD/GND adolescents were more likely than boys to have accessed any support (v/n) as well as multiple types of support (e/v). A total of 33.4% (4090/12233) of girls reported past-year access to mental health support, with 59.1% (2419/4090) of these reporting accessing multiple types; for GD/GND participants these proportions were 41.2% (437/1061) and 60.6% (265/437), respectively; and for boys 17.9% (1865/10419) and 50.6% (944/1865), respectively.

Across genders, patterns of support accessed generally mirrored the overall network, with informal and school-based supports most commonly accessed. However, the networks demonstrate marked gender differences in the number of different supports and perceived helpfulness. Although boys were less likely to seek support, when they did, they generally perceived it as helpful. Girls perceived most types of support as slightly less helpful, particularly for formal and semi-formal supports (e.g. only 54.5% of girls found CAMHS helpful, compared with 68.8% of boys). GD/GND individuals had the densest network in terms of accessing multiple types of support and showed substantial differences in terms of perceived helpfulness compared with boys and girls. Whilst informal supports (particularly friends both in-person and friends online) and certain semi-formal supports (namely charities and other school adults) were still generally perceived as helpful, helpfulness of formal and most other semi-formal supports was strikingly low. For example, amongst GD/GND participants, 40.0% found CAMHS helpful, 56.3% found school mental health teams helpful, 54.5% found online support helpful, and 46.7% found helpline support helpful.

**Figure 5.**
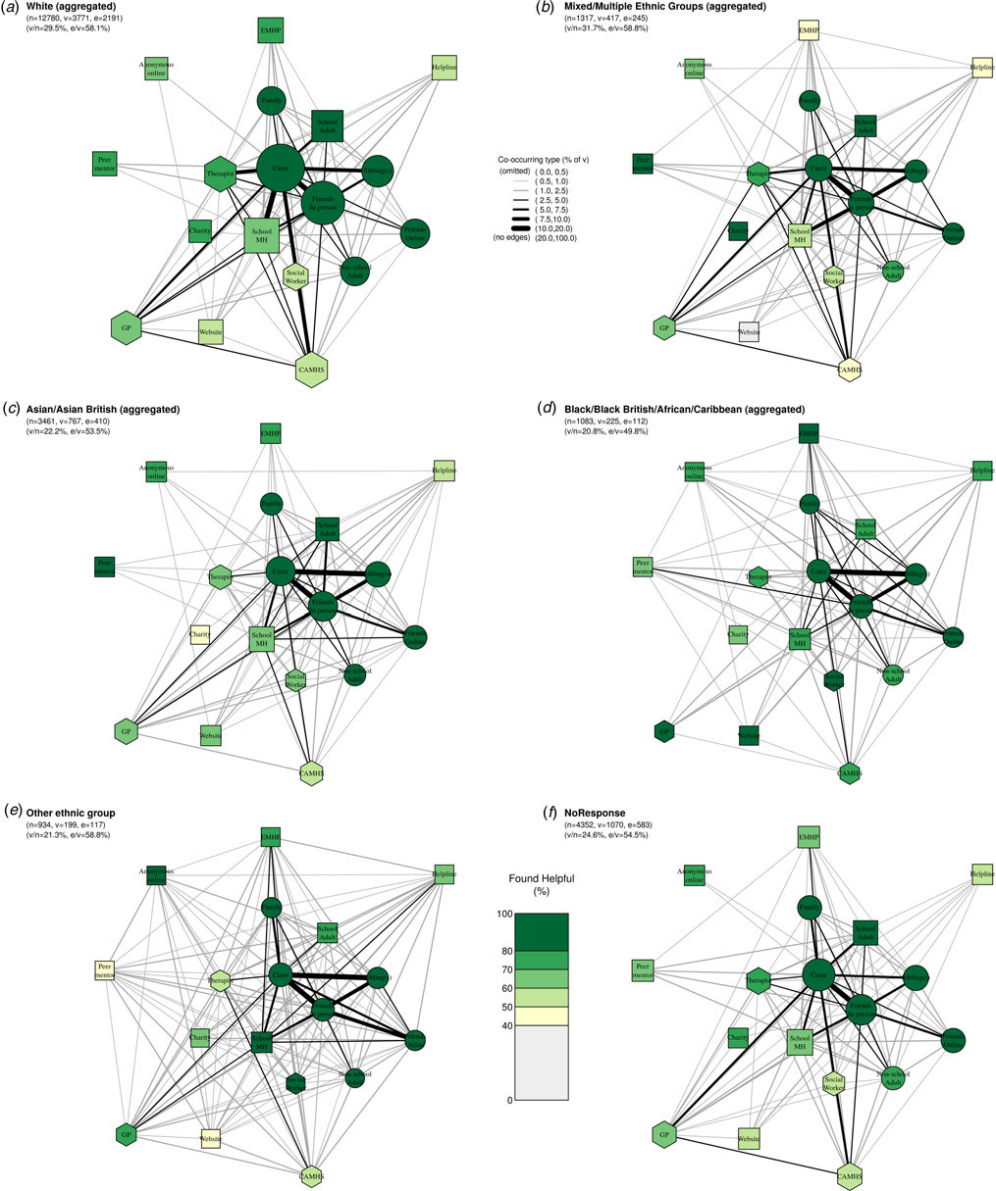
**Networks of care by self-reported ethnicity: (a) White (aggregated); (b) Mixed/Multiple Ethnic Groups; (c) Asian/Asian British; (d) Black/Black British/African/Caribbean; (e) Other Ethnic Group; (f) ethnicity not reported.** Within each network, node size corresponds to the absolute number of participants who accessed each type of support in the past year; edge thickness corresponds to the proportion of participants in the respective subgroup whoaccessed both types of support; node colour corresponds to the proportion of participants whofound each type of support helpful; and nodeshape corresponds to our grouping of support types where circles are informal supports, squares semi-formal, and hexagons formal. Legend for short labels: Carer: parent, step-parent, or carer; Sibling: sibling(s); Family: someone else in your family; Friends In Person: friend(s), mainly known in person; Friends Online: friend(s), mainly known online; Other Adult: an adult outside of school (at a sport club, another parent, family friend); School MH: school nurse/counsellor/other pastoral staff at school; EMHP: Education Mental Health Practitioner; School Adult: another adult at school; Peer: a peer mentor at school; Charity: support service given by a charity; Helpline: a telephone/text helpline; Website: website or online forum; Anonymous Online: an anonymous user on an online platform/chatroom/forum/server; GP: GP (family doctor); Social worker: social worker; CAMHS: NHS Child and Adolescent Mental Health Services; Therapist: private counsellor/therapist.

Figure 5 presents networks stratified by ethnicity. The proportion reporting past-year access to mental health support was lowest amongst Black/Black British/African/Caribbean adolescents (20.8%, 225/1083), Asian/Asian British adolescents (22.2%, 767/3461), and those from ‘Other’ ethnic groups (21.3%, 199/934) and was highest amongst White adolescents (29.5%, 3771/12780) and adolescents from Mixed/Multiple ethnic groups (31.7%, 417/1317).

Across these networks, some nodes and edges have very few individuals contributing to them, so caution is needed when comparing across groups. However, general patterns of the overall network (Fig. 2) held across ethnic groups, whereby informal support was the most commonly accessed and perceived to be most helpful, followed by semi-formal and then formal support. For adolescents from Black/Black British/African/Caribbean, Asian/Asian British, and ‘Other’ ethnic groups in particular, the sub-network formed by parents and carers, siblings, and friends in-person seemed especially important (as demonstrated by the edges connecting these types of support).

Additional networks by year groupings (online Supplementary Fig. S3) and self-identified past-year mental health problems (online Supplementary Fig. S4) are presented in the online Supplementary Material.

### Anchored networks

**Figure 6.**
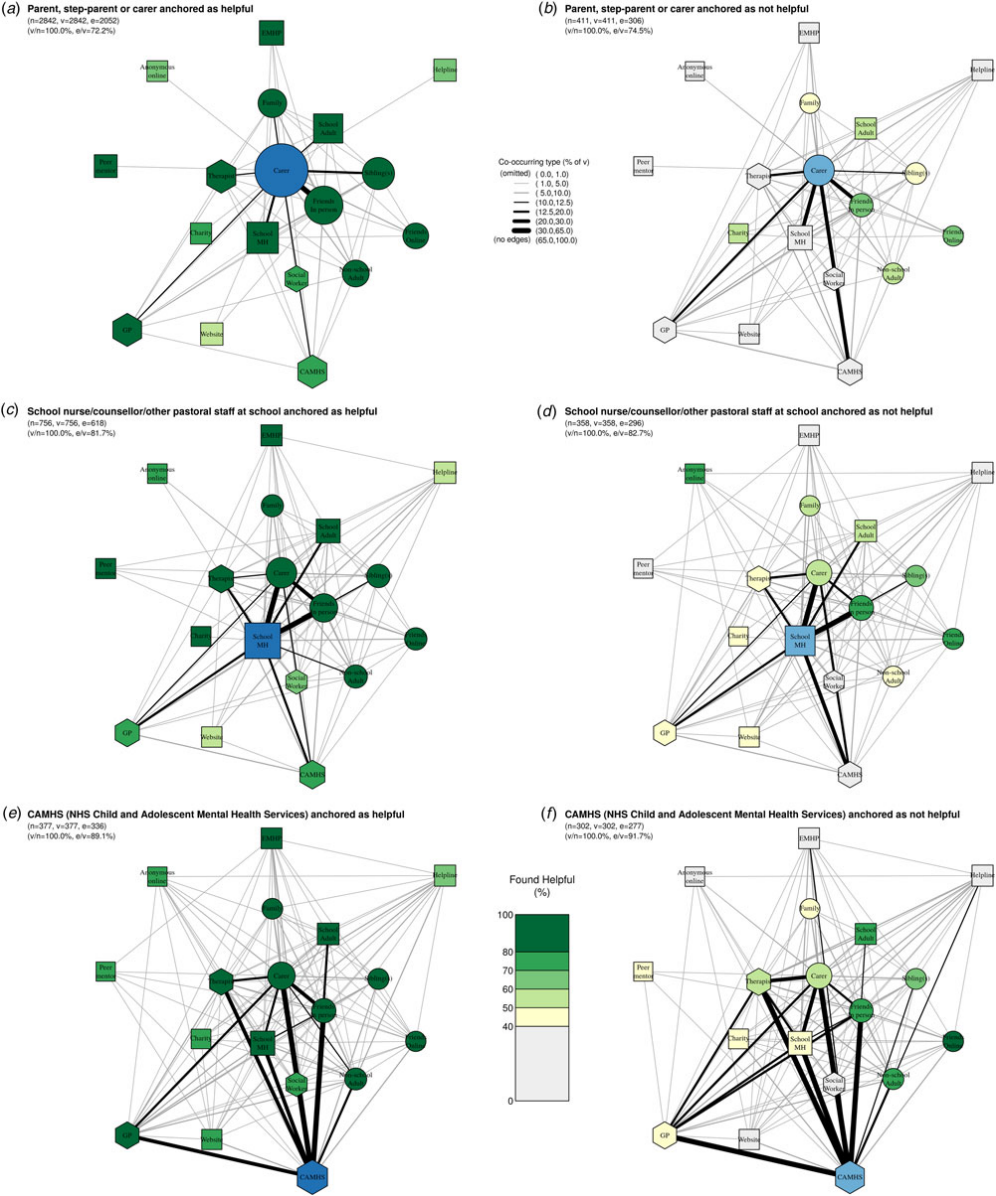
**Networks of care for ‘anchored pairs’.** Pairs represent those who did (dark blue) and did not (light blue) find three key types of support helpful: parents/carers (a/b), school mental health teams (including school nurses/counsellors/other pastoral staff) (c/d), and CAMHS (e/f). Within each network, node size corresponds to the absolute number of participants who accessed each type of support in the past year; edge thickness corresponds to the proportion of participants in the respective subgroup who accessed both types of support; node colour corresponds to the proportion of participants who found each type of support helpful; and node shape corresponds to our grouping of support types where circles are informal supports, squares semi-formal, and hexagons formal. Legend for short labels: Carer: parent, step-parent, or carer; Sibling: sibling(s); Family: someone else in your family; Friends In Person: friend(s), mainly known in person; Friends Online: friend(s), mainly known online; Other Adult: an adult outside of school (at a sport club, another parent, family friend); School MH: school nurse/counsellor/other pastoral staff at school; EMHP: Education Mental Health Practitioner; School Adult: another adult at school; Peer: a peer mentor at school; Charity: support service given by a charity; Helpline: a telephone/text helpline; Website: website or online forum; Anonymous Online: an anonymous user on an online platform/chatroom/forum/server; GP: GP (family doctor); Social worker: social worker; CAMHS: NHS Child and Adolescent Mental Health Services; Therapist: private counsellor/therapist.

Figure 6 presents ‘anchored’ network pairs illustrating differences according to the perceived helpfulness of a focal support type. Figure 6(a) shows the network for those who had accessed support from a parent/carer and found it helpful, whilst Fig. 6(b) shows the network for those who had accessed support from their parent/carer but *not* found it helpful. Figure 6(c/d) shows analogous networks for school mental health teams and Fig. 6(e/f) for CAMHS.

These networks demonstrate a general trend that if an adolescent finds one of these three ‘key’ supports helpful (Figs 6(a), (c), and (e)), they are more likely to find other types of support helpful as well, whereas if they *do not* find the key support helpful (Figs 6(b), (d), and (f)), the opposite is true. As the ‘anchored’ category of support moves from informal (parent/carer) to semi-formal (school mental health team) to formal (CAMHS), the networks become denser, suggesting that adolescents access more types of support as they move into more formal support systems.

## Discussion

The networks of care elucidated by our diverse school-based sample highlight the myriad ways today's adolescents are seeking support in one of the most challenging areas of care – that of mental health provision. Services need to understand how adolescents approach and perceive informal, semi-formal, and formal providers to better tailor care offered. This is especially pertinent for those populations with the greatest need and those finding it difficult to access support or perceiving care received as unhelpful. For the one-in-four adolescents who reported past-year support, informal support from friends and family was most central and perceived as most helpful, including for those with elevated symptoms of depression and anxiety. Girls and GD/GND adolescents were generally more likely to access (multiple types of) support and to find support less helpful than boys. There was variation by ethnicity in terms of the proportion accessing support, but general patterns mirrored those of the full sample, with informal support being most central and perceived as most helpful. The networks also enabled us to examine key groups, such as those who had received mental health support from key adults but had not found them helpful. These ‘anchored’ networks highlighted the isolation of adolescents who did not perceive support from their parents/carers as helpful, as they tended to find little else helpful either.

For most individuals, informal networks are a mainstay of care and connection and often the first port of call for providing or facilitating access to mental health support (Lynch, Moorhead, Long, & Hawthorne-Steele, 2023; Mandalia et al., 2018). Our findings reinforce the importance of these networks. Whilst these relationships have supported individuals for millennia, it is important from a public mental health lens to identify when these authentic, organic systems might come under pressure and could benefit from additional scaffolding. Current stressors, including strain on global economic systems and associated inequalities, are placing sustained pressure on family systems, which may in turn impact on adolescent mental health (Kirkbride et al., 2024). For example, financial pressures might take caregivers away from the nuclear home for longer periods of time as well as negatively impact on time and ability to invest into community systems and structures that might otherwise be a key source of support for adolescents. Schools might have to take difficult decisions between, for example, investing in supports for specific students versus broader pastoral and social activities that can underpin opportunities to build authentic social networks and promote school belonging for all students.

In considering the implications of these networks of care for formal systems of support such as mental health services and social care provision, it is clear that many adolescents are seeking help in multiple places, especially when they reach the threshold of needing to access more formal, institutional sources of support. This highlights how services need to work in concert with one another, as whilst some models of provision support a more integrated approach, others struggle to achieve this, particularly when physical locations, structures, and systems can make it difficult to collaborate in shared care provision, therapeutic approaches, and monitoring arrangements, which is further exacerbated by time, data-sharing, and financial constraints (Farr et al., 2021; Fazel et al., 2021b; Fazel, Rocks, Glogowska, Stepney, & Tsiachristas, 2021a; Wolfe, Satherley, Scotney, Newham, & Lingam, 2020).

On discussing these networks with a YPAG of eight 17–18-year-olds, their conclusions focused on the importance of those adolescents who seemed most isolated and did not perceive support from their parents/carers as helpful. The importance of the broader network of adults external to formal services and structures, such as adults known via extracurricular activities and charities, seemed key. However, access to these opportunities often depends on factors outside of an adolescent's control, which might mean that not all have an equal chance to make and benefit from these connections. Reflecting the wider literature (e.g. Radez et al., 2020), our YPAG also explained how central trust can be in seeking mental health support and the difficulty of establishing that with someone who is a ‘stranger’ to them, further underscoring the benefits of informal and semi-formal connections.

By examining mental health support from the perspective of young people themselves, our networks help us better understand how adolescents interact with the complex system of services and supports available to them. OxWell's scale, coverage, and anonymity have allowed us to explore the views of adolescents whose perspectives and experiences are not captured in other ways. This is particularly relevant for adolescents from vulnerable or marginalized groups, who have distinct experiences of mental health services (Mannes et al., 2024; Soneson et al., 2024), but whose views are often underexplored in research. Combined with data from other sources, such as qualitative studies and youth advisory groups, these survey data can help us better capture young people's perspectives. Further triangulation through stakeholder involvement with families and those working across semi-formal and formal services can help provide a more complete picture of the complex implementation landscape in child and adolescent mental health (Clarke & Barwick, 2021; Zolfaghari et al., 2022).

To this end, understanding the range of potential supports can help to further contextualize the network findings, as whilst statutory services (e.g. CAMHS and social care) are relatively consistent across regions of England (including our study sites), there is substantial variation both across and within regions with regards to what types of mental health support is on offer in schools (Marshall, Wishart, Dunatchik, & Smith, 2017) and the wider community. Characterization of these supports can help answer questions pertaining to constructs of *availability* and, to some extent, *accessibility*, in addition to *acceptability*. Expansion of the concept of ‘acceptability’ could also be fruitful, given the many complexities of understanding what it means for support to be ‘helpful’. It remains necessary, for example, to consider the impact of underlying psychopathology on an individual's perceived need for support, ability to seek support, and perceptions of support received. An adolescent suffering from a moderate to severe depressive disorder might not perceive any support as ‘helpful’, despite that support being a necessary component of their treatment that might not be recognized until after treatment or maybe ever. Therefore, it cannot be assumed that ‘not helpful’ equates to poor quality, although the sheer number who report not finding CAMHS helpful does merit further examination, ideally utilizing mixed methods, multiple informants, and longitudinal studies. Therefore, exploration of interrelated constructs, such as expectations of and satisfaction with support (Børge & Yngvild, 2024), may help understand young people's perspectives on support received as well as the impact it has had on them and their wider social networks.

### Strengths and limitations

Our network approach enabled us to begin to quantitatively examine the full complexity of how the modern adolescent navigates the system of mental health services and supports available to them and elucidate the interconnections between different sources of support. The expanded view on what constitutes ‘mental health support’ is another key strength; most previous studies have been centered around formal mental health services, relatively few have further evaluated access to and perceptions of school-based services, and even fewer have examined informal sources of support. Shifting the lens of enquiry to this broader view of support enabled us to gain new insight into adolescent help-seeking. Furthermore, our data were collected directly from adolescents, reflecting the critical importance of the youth voice for service design and evaluation (Fazel & Hoagwood, 2021; Plaistow et al., 2014). The inclusion of perceived helpfulness highlighted groups with less positive perceptions of key sources of support and thus provided insight regarding potential ways to enhance support overall. Finally, the use of a large school-based, community sample helped us to address some of the common sampling biases in the assessment of adolescents' perceptions of mental health services.

Our findings should also be contextualized within a number of limitations. Due to the recruitment strategy used (Mansfield et al., 2021), we could not determine the participation rate, which limits our ability to fully assess potential selection bias. Study methodology (including that socioeconomic status was not assessed at an individual level) and lack of appropriate reference data also make it difficult to determine whether the sample is representative of its target population; however, the sample was diverse across a range of characteristics including ethnicity, geographic area, type of school attended, and area-level socioeconomic deprivation characteristics of the school, suggesting that we captured a broad range of perspectives and experiences. Importantly, there are several groups not represented and underrepresented in our sample who may interact with mental health services and support in systematically different ways, including students at non-mainstream schools, students absent from school on the day of survey administration, and students present but who could not – or chose not to – engage with the survey for various reasons.

Regarding data analysed, access to support was collected through self-report, with limited context as to what types of support were available in each student's school and wider area. However, although this may have introduced bias, there are also substantial benefits of self-report, namely that it enabled exploration of informal and semi-formal support often not captured in routine data. Outcome ascertainment may also have been influenced by the structure of the questionnaire, as it is possible that the relatively high prevalence of informal support (presented first, followed by semi-formal and formal support) was due in part to adolescents becoming increasingly disengaged with the many answer options. In terms of acceptability, we asked about whether support accessed was ‘helpful’, without further description for participants of what we meant by ‘helpful’, and this one question informed our conclusions on acceptability of service provision. In addition, within each of the 18 different support options, there was no opportunity to disaggregate further, which complicates interpretation of ‘helpfulness’. If, for example, a student sought help from multiple other adults at school, this was not captured in the survey, and if the student found some adults helpful and others less so, it is unclear how this question would therefore have been answered. In terms of study design, the cross-sectional nature of the data prohibits study of temporal trends in the networks. Finally, in terms methodological approach, we do not perform a direct quantitative assessment of network differences. Since a crude omnibus test of ‘no difference’ between networks is likely to be trivially rejected, and at present there is lack of clarity as to which specific statistical test is required to determine which features of the network vary, and to what extent.

## Conclusion

Today's adolescents exist within a rapidly evolving world of interconnected interpersonal, community, and institutional arenas, each of which offers myriad potential opportunities to access mental health support, if needed (Fazel & Soneson, 2023). Examining interrelationships of help-seeking pathways and preferences across these networks can inform ways to design provision according to different needs across different spaces. Our analysis of the OxWell Student Survey highlights the multiple ways adolescents are seeking support across informal, semi-formal, and formal systems, raising important and interesting issues regarding which type(s) of support are accessed and by whom, as well as about groups for whom existing support systems might not be sufficiently meeting their needs. Services can no longer be designed, developed, delivered, or measured in isolation from the networks the modern adolescent inhabits. Young people need to be involved in determining how best to ask about the acceptability of the support they seek, with these questions at the heart of any service evaluations.

## Supporting information

White et al. supplementary material 1 White et al. supplementary material

White et al. supplementary material 2 White et al. supplementary material

## Data Availability

All authors had full access to all the data in the study and accept responsibility for submitting the paper for publication. Fully deidentified extracts of the data can be provided to academic research collaborators upon reasonable request after a review process by the research team to ensure that uses of the data fall under the remit of the intended purposes set out in the privacy information and to prevent duplication of analyses. The data are not publicly available because of ethical and information governance restrictions. The full list of questions as well as other details are available on a project-specific OxWell Open Science Framework page (https://osf.io/sekhr/).

## Supplementary material

The supplementary material for this article can be found at https://doi.org/10.1017/S003329172400237X

## Code availability

Code to perform the hurdle model analysis and create all plots is publicly available via https://github.com/simon-r-white/networks_of_care. All analysis was performed using GNU R v4.1.2.
