# Supplementary material for: Networks of care for the modern adolescent
Source: Psychol Med. 2024 Dec 19;54(16):4537–50. doi: 10.1017/S003329172400237X (PMC11769907; doi:10.1017/S003329172400237X)
Supplement: White et al. supplementary material 1 — White et al. supplementary material [file S003329172400237Xsup001.docx]

# Supplementary material


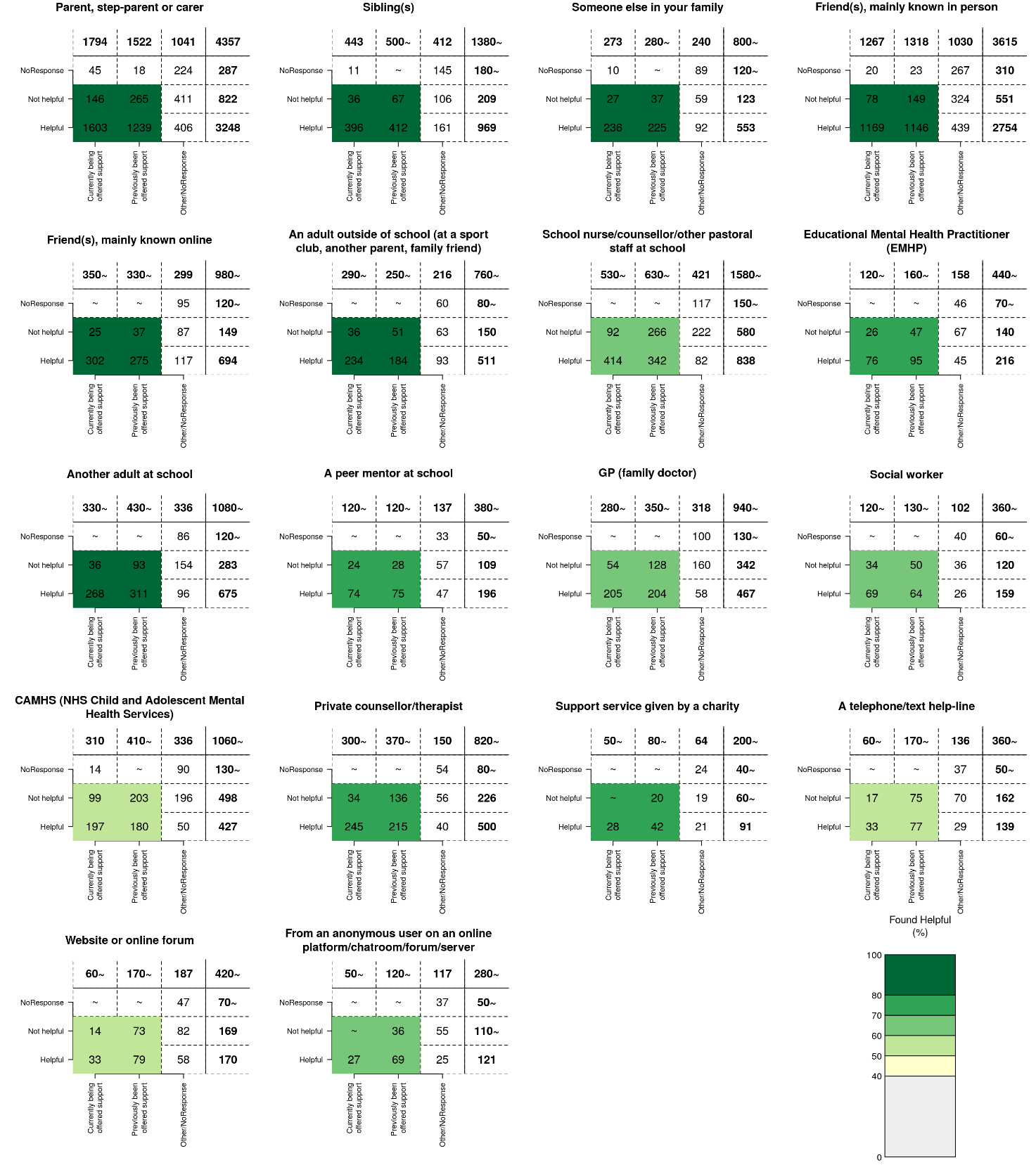


**Fig S1. | Numbers of participants reporting having sought each type of mental health support in the previous year and the associated perceived helpfulness of those supports.** Plots include non-response for helpfulness, and status includes a column for those who sought, but did not access, the support in the past year, i.e. those excluded from our analyses. The proportion of participants who found the service helpful is shown in green (note this proportion is calculated using the highlighted four cells only). Counts less than ten (and associated totals) are censored, denoted with a tilde.

***
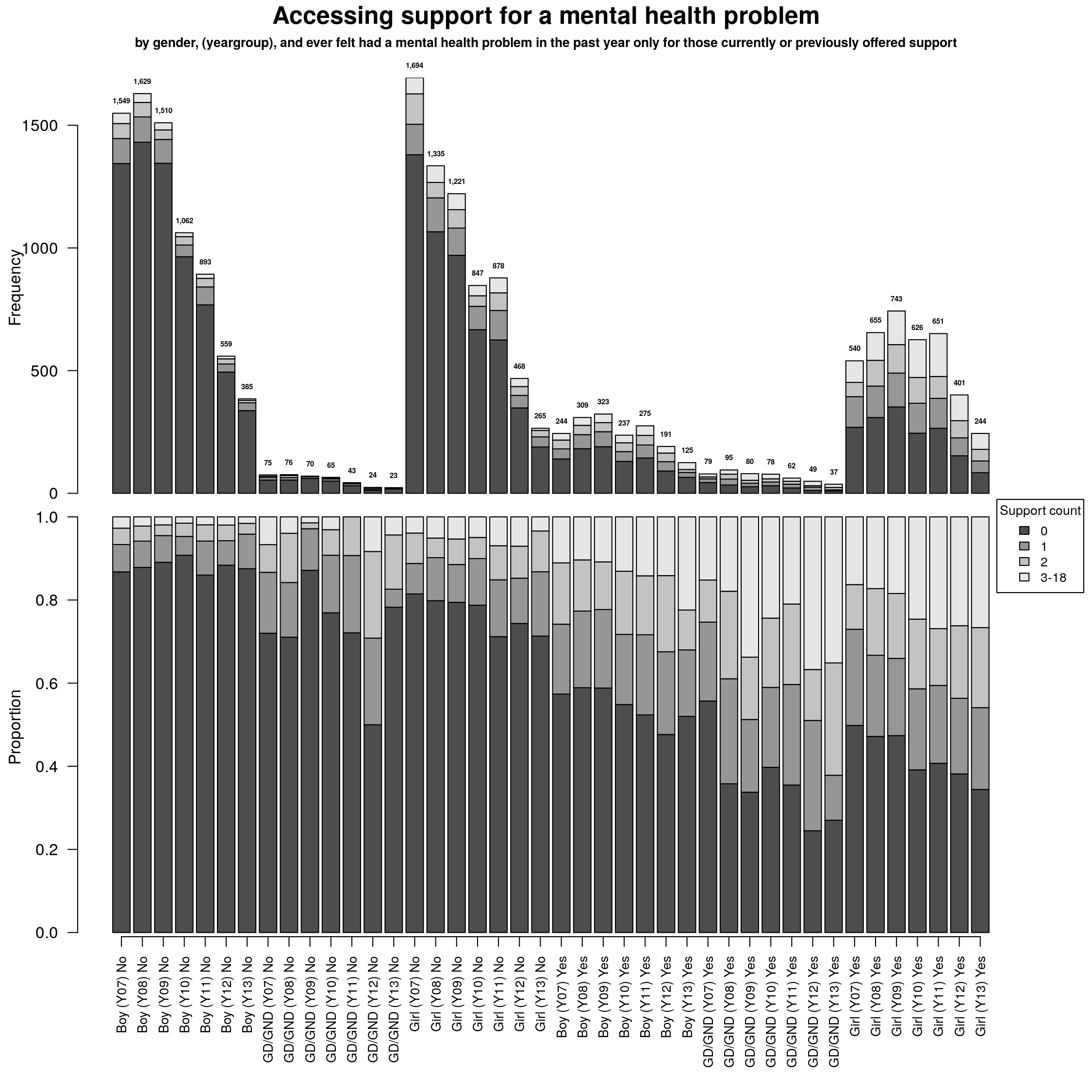
***

**Fig S2**. | **Extent of multiple access to different types of mental health support according to gender, year group, and self-identified mental health difficulties; reported as (a)** **counts and (b) proportions.** *Note: 3,041 participants with unknown self-identified past-year mental health difficulties are omitted (see Table 1).*


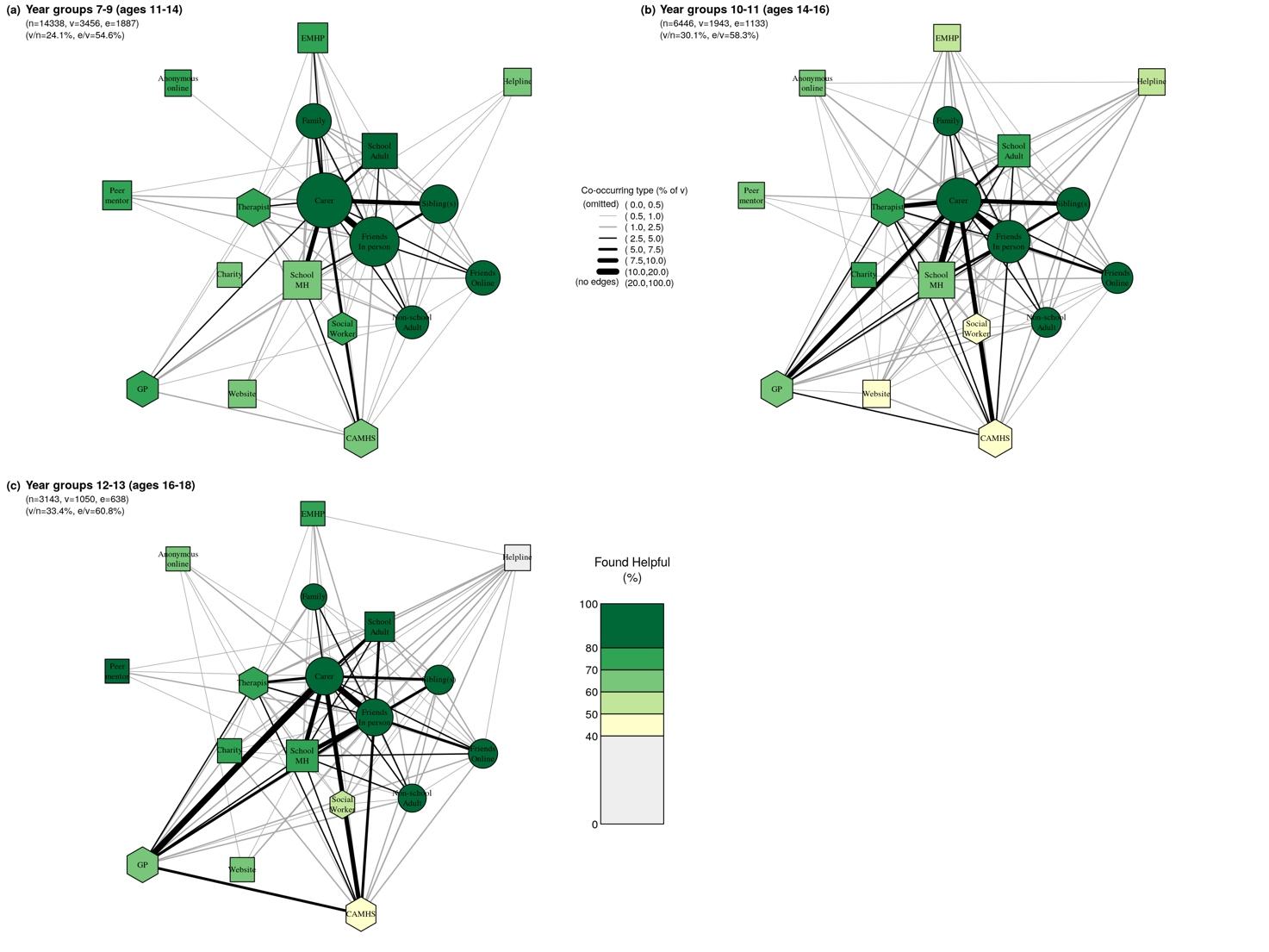


**Fig S3. | Networks of care for students in year groups (a) 7-9 (ages 11-14 years); (b) 10-11 (ages 14-16 years); (c) 12-13 (ages 16-18 years).** *Within each network, node size corresponds to the absolute number of participants who accessed each type of support in the past year; edge thickness corresponds to the proportion of participants in the respective subgroup who accessed both types of support; node colour corresponds to the proportion of participants who found each type of support helpful; and node shape corresponds to our grouping of support types where circles are informal supports, squares semi-formal, and hexagons formal. Legend for short labels:* ***Carer:*** *Parent, step-parent, or carer;* ***Sibling****: Sibling(s);* ***Family****:* *Someone else in your family);* ***Friends In Person****: Friend(s), mainly known in person;* ***Friends Online****:* *Friend(s), mainly known online;* ***Other Adult****:* *An adult outside of school (at a sport club, another parent, family friend);* ***School MH****: School nurse/counsellor/other pastoral staff at school;* ***EMHP****: Education Mental Health Practitioner;* ***School Adult:*** *Another adult at school;* ***Peer****: A peer mentor at school;* ***Charity****: Support service given by a charity;* ***Helpline:*** *A telephone/text helpline;* ***Website:*** *Website or online forum;* ***Anonymous Online:*** *An anonymous user on an online platform/chatroom/forum/server;* ***GP:*** *GP (family doctor);* ***Social worker****: Social worker;* ***CAMHS:*** *NHS Child and Adolescent Mental Health Services);* ***Therapist****: Private counsellor/therapist.*

**
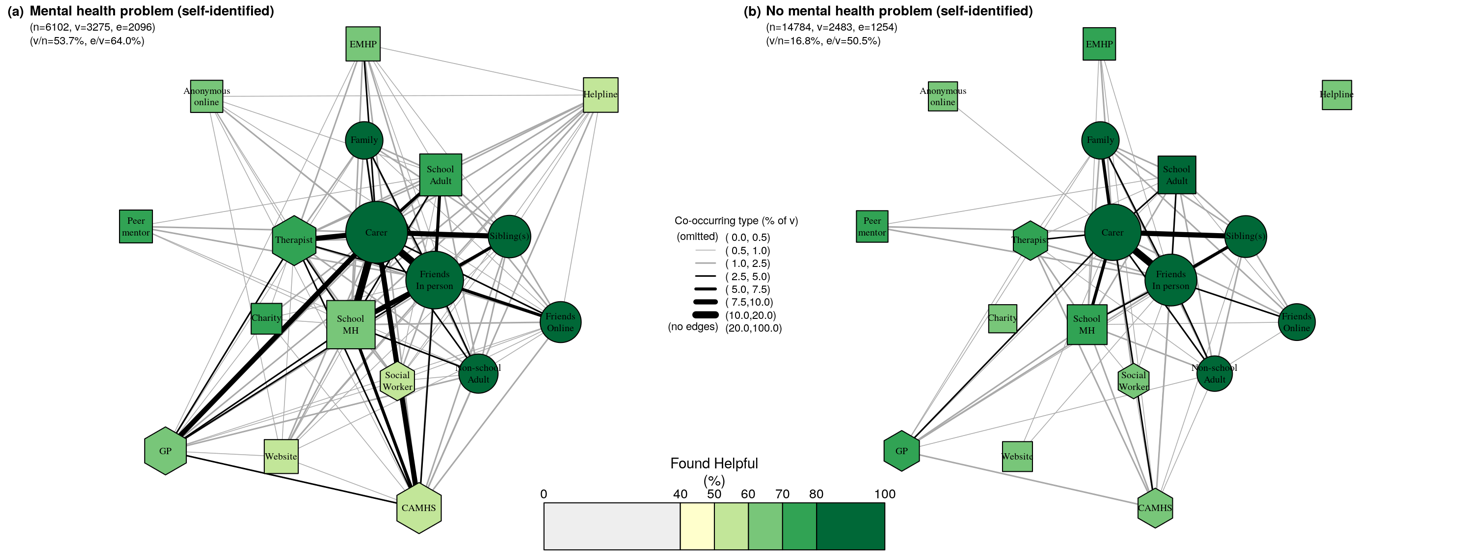
Fig S4. | Networks of care for students (a) with self-identified past-year mental health problems and (b) without self-identified past-year mental health problems.** *Within each network, node size corresponds to the absolute number of participants who accessed each type of support in the past year; edge thickness corresponds to the proportion of participants in the respective subgroup who accessed both types of support; node colour corresponds to the proportion of participants who found each type of support helpful; and node shape corresponds to our grouping of support types where circles are informal supports, squares semi-formal, and hexagons formal. Legend for short labels:* ***Carer:*** *Parent, step-parent, or carer;* ***Sibling****: Sibling(s);* ***Family****:* *Someone else in your family);* ***Friends In Person****: Friend(s), mainly known in person;* ***Friends Online****:* *Friend(s), mainly known online;* ***Other Adult****:* *An adult outside of school (at a sport club, another parent, family friend);* ***School MH****: School nurse/counsellor/other pastoral staff at school;* ***EMHP****: Education Mental Health Practitioner;* ***School Adult:*** *Another adult at school;* ***Peer****: A peer mentor at school;* ***Charity****: Support service given by a charity;* ***Helpline:*** *A telephone/text helpline;* ***Website:*** *Website or online forum;* ***Anonymous Online:*** *An anonymous user on an online platform/chatroom/forum/server;* ***GP:*** *GP (family doctor);* ***Social worker****: Social worker;* ***CAMHS:*** *NHS Child and Adolescent Mental Health Services);* ***Therapist****: Private counsellor/therapist.*

**Table S1 | Numbers of participants who likely did not reach the questions asking about mental health support in the OxWell Student Survey**

Notes: (a) For participant self-reported gender we have combined the gender diverse (GD) category with the gender non-disclosing (GND) category (see Methods); (b) we report ethnicity by ONS ethnic group classification 6a; (c) starting the survey was dependent on the year group question being answered, and so there is no missing data for this question; (d) we present mean-imputed RCADS-11 scores, allowing for up to two missing items (≤1 per sub-scale); (e) the RCADS-11 only has clinical thresholds for boys and girls; we have used a threshold of ≥14 for GD/GND participants; (f) we have excluded those who are unknown for gender and/or RCADS-11 (as per point (d) above).

| **Non-responder descriptive** | | **Total** |
| --- | --- | --- |
| All |  | 5,344 |
| Gender | Boy | 2,857 |
|  | Girl | 2,173 |
|  | GD/GND^a^ | 221 |
|  | No response | 93 |
| Ethnicity | Asian/Asian British | 613 |
|  | Black/Black British/African/Caribbean | 340 |
|  | Mixed/Multiple ethnic groups | 332 |
|  | White | 2,675 |
|  | Other ethnic group | 286 |
|  | No response | 1,098 |
| Year group | Y07 | 1,305 |
|  | Y08 | 927 |
|  | Y09 | 910 |
|  | Y10 | 820 |
|  | Y11 | 649 |
|  | Y12 | 456 |
|  | Y13 | 277 |
|  | Unknown year group^c^ | 0 |
| Gender & RCADS-11^d^ | Normal | 883 |
|  | Boy, clinical (≥9) | 278 |
|  | Girl, clinical (≥14) | 285 |
|  | GD/GND, clinical (≥14)^e^ | 28 |
|  | Unknown gender and/or RCADS-11^f^ | 3,870 |

**Table S2 | Hurdle model coefficients.** *Coefficients for the binomial and Poisson components of the hurdle model used to assess the pattern seen in Table 1 (main text). Hurdle models are non-trivial to interpret from the coefficients directly, hence we present the model graphically in Figure 1 (main text).*

|  | currently or previously offered support | | | | |  |
| --- | --- | --- | --- | --- | --- | --- |
| *Predictors* | *Log-Mean* | *CI* | | *p* |  | |
| **Count Model** | | | | | |  |
| (Intercept) | 0.15 | 0.06 – 0.24 | | 0.001 |  | |
| Gender [GD/GND] | -0.01 | -0.18 – 0.16 | | 0.871 |  | |
| Gender [Girl] | 0.14 | 0.05 – 0.24 | | 0.002 |  | |
| Year group | 0.01 | -0.02 – 0.03 | | 0.454 |  | |
| RCADS-11 [Clinical] | 0.09 | 0.01 – 0.17 | | 0.020 |  | |
| Self-identified MH difficulties [Yes] | 0.25 | 0.17 – 0.33 | | <0.001 |  | |
| Gender [GD/GND] × Year group | 0.08 | 0.03 – 0.12 | | 0.003 |  | |
| Gender [Girl] × Year group | 0.03 | -0.00 – 0.06 | | 0.069 |  | |
| RCADS-11 [Clinical] × Self-identified MH difficulties [Yes] | 0.08 | -0.03 – 0.18 | | 0.147 |  | |
| **Zero-Inflated Model** | | |  |  |  |  |
| (Intercept) | -2.34 | -2.44 – -2.23 | | <0.001 |  | |
| Gender [GD/GND] | 0.58 | 0.31 – 0.84 | | <0.001 |  | |
| Gender [Girl] | 0.49 | 0.37 – 0.61 | | <0.001 |  | |
| Year group | 0.01 | -0.02 – 0.04 | | 0.477 |  | |
| RCADS-11 [Clinical] | 1.15 | 1.06 – 1.24 | | <0.001 |  | |
| Self-identified MH difficulties [Yes] | 1.79 | 1.68 – 1.91 | | <0.001 |  | |
| Gender [GD/GND] × Year group | 0.10 | 0.01 – 0.19 | | 0.030 |  | |
| Gender [Girl] × Year group | 0.08 | 0.04 – 0.12 | | <0.001 |  | |
| RCADS-11 [Clinical] × Self-identified MH difficulties [Yes] | -0.92 | -1.07 – -0.77 | | <0.001 |  | |
| Observations | 20207 | | | | |  |
| R2 / R2 adjusted | 0.182 / 0.181 | | | | |  |

**Table S3 | Helpfulness (percentage) across support types for all participants and by gender.** Helpfulness percentage is calculated excluding missing data, see Supplementary Figure 1 for a visual representation (for example across all participants, 87.4% = (1603+1239)/(1603+1239+146+265) found their parent/carer helpful). Note: see Figure 2 (main text) to map short label names to types of support; table ordered based on helpful percentage across all participants.

|  | **All** | **Boy** | **GD/GND** | **Girl** |
| --- | --- | --- | --- | --- |
| Friends in person | 91.1 | 92.1 | 88.3 | 91.3 |
| Friends online | 90.3 | 91.5 | 88.8 | 90.6 |
| Sibling(s) | 88.7 | 86.6 | 86.7 | 90.1 |
| Family | 87.8 | 87.0 | 75.9 | 89.4 |
| Carer | 87.4 | 91.4 | 78.5 | 86.3 |
| Non-school adult | 82.8 | 84.4 | 88.6 | 81.6 |
| School adult | 81.8 | 82.6 | 76.1 | 82.1 |
| Peer mentor | 74.1 | 74.7 | 70.6 | 77.5 |
| Therapist | 73.0 | 81.7 | 78.7 | 70.6 |
| Charity | 70.7 | 72.7 | 88.9 | 69.2 |
| EMHP | 70.1 | 80.7 | 50.0 | 65.8 |
| GP | 69.2 | 78.2 | 65.9 | 67.2 |
| Anonymous online | 68.6 | 76.3 | 66.7 | 67.9 |
| School MH | 67.9 | 76.2 | 56.2 | 66.7 |
| Social worker | 61.3 | 66.2 | 54.5 | 59.6 |
| Website | 56.3 | 69.2 | 54.5 | 52.9 |
| CAMHS | 55.5 | 68.8 | 40.0 | 54.5 |
| Helpline | 54.5 | 62.5 | 46.7 | 53.9 |
